# Supplementary material for: Correlation of Neutralizing Antibodies (NAbs) between Sows and Piglets and Evaluation of Protectability Associated with Maternally Derived NAbs in Pigs against Circulating Porcine Reproductive and Respiratory Syndrome Virus (PRRSV) under Field Conditions
Source: Vaccines (Basel). 2021 Apr 21;9(5):414. doi: 10.3390/vaccines9050414 (PMC8143086; doi:10.3390/vaccines9050414)
Supplement: Supplementary file 1 [file vaccines-09-00414-s001.zip › vaccines-1178842-supplementary.pdf]

Supplementary Materials

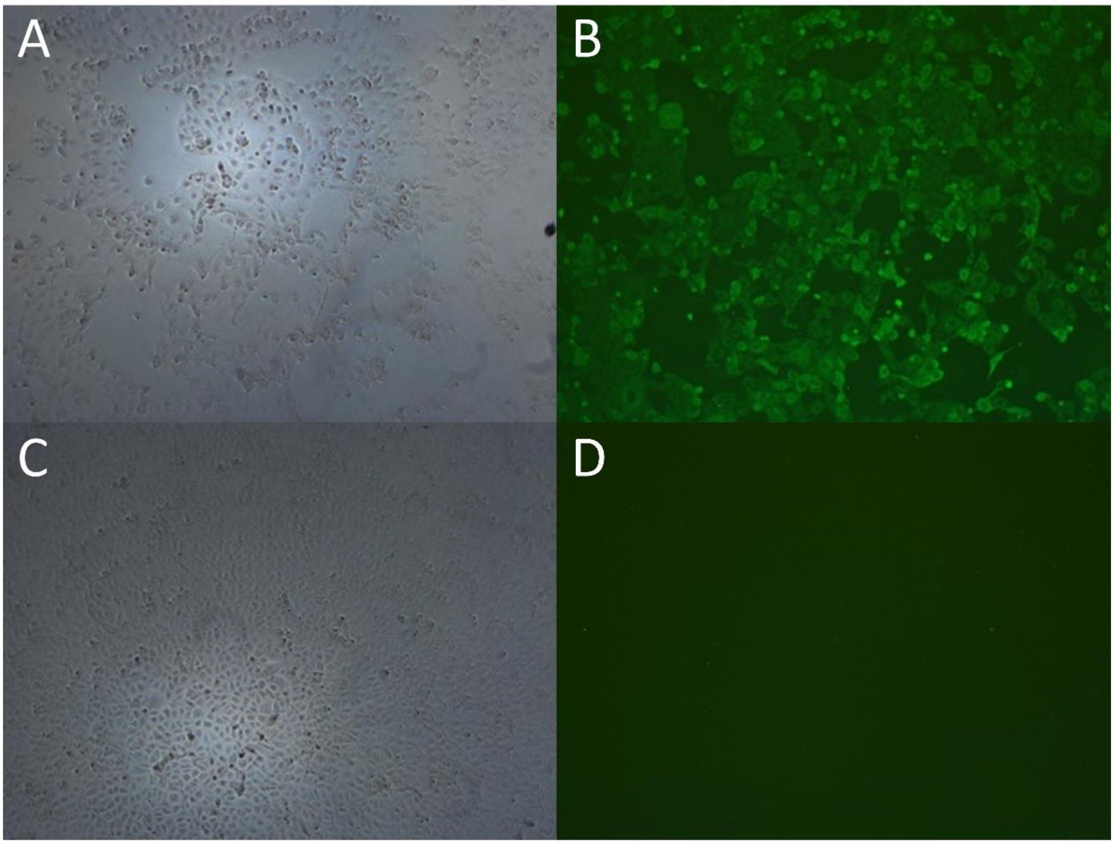

Figure S1. Immunofluorescence assay (IFA) of PRRSV in MARC145 cells for viral neutralization assay. Cytopathic effects (CPE) of infected MARC145 cells were visualized (A) and stained by IFA utilizing PRRSV monoclonal antibodies and fluorescein goat anti-mouse secondary antibodies (B). The naïve parental MARC145 cells were presented (C) and examined by IFA using the same procedure (D). All pictures were recorded under ×50 magnification.
